# In renal proximal tubular epithelial cells of the hibernator Syrian hamster, anoxia-reoxygenation-induced reactive oxygen species bursts do not trigger a DNA damage response and cellular senescence

**DOI:** 10.1007/s00360-025-01604-5

**Published:** 2025-02-05

**Authors:** Georgios Pissas, Maria Divani, Maria Tziastoudi, Christina Poulianiti, Maria-Anna Polyzou-Konsta, Evangelos Lykotsetas, Ioannis Stefanidis, Theodoros Eleftheriadis

**Affiliations:** Department of Nephrology, Faculty of Medicine, University of Thessaly, Biopolis, Mezourlo Hill, Larissa, 41110 Greece

**Keywords:** Ischemia-reperfusion, Acute kidney injury, Hibernation, DNA damage response, Senescence

## Abstract

Ischemia-reperfusion (I-R) injury represents a predominant etiology of acute kidney injury (AKI), for which effective treatments remain unavailable. In contrast, hibernating mammals exhibit notable resistance to cell death induced by I-R injury. However, the impact of I-R injury on cellular senescence—an important factor in AKI—has not been extensively studied in these species. Comparative biology may offer novel therapeutic insights. Renal proximal tubular epithelial cells (RPTECs) from the native hibernator Syrian hamster or mouse RPTECs were subjected to anoxia-reoxygenation. Proteins involved in DNA damage response (DDR) and cellular senescence were assessed using western blotting, reactive oxygen species (ROS) levels and cell death were quantified colorimetrically, and IL-6 with ELISA. Anoxia-reoxygenation induced oxidative stress in both mouse and hamster RPTECs; however, cell death was observed exclusively in mouse cells. While anoxia-reoxygenation elicited a DDR and subsequent senescence in mouse RPTECs, such responses were not detected in hamster RPTECs. Thus, RPTECs from the Syrian hamster exhibited increased ROS production upon reoxygenation but did not show DDR or cellular senescence. Further research is required to elucidate the specific protective molecular mechanisms in hibernators, which could potentially lead to the development of novel therapeutic approaches for I-R injury in non-hibernating species, including humans.

**Supplementary Information:**

The online version contains supplementary material available at 10.1007/s00360-025-01604-5.

## Introduction

Ischemia-reperfusion (I-R) injury is a critical factor in the pathogenesis of numerous diseases, including acute kidney injury (AKI). I-R injury occurs when blood supply is reduced or halted due to conditions such as various types of shock, decreased effective blood volume, or arterial occlusion (Neri et al. 2017; Bakthavachalam and Shanmugam 2017; Tsukamoto et al. 2010; Bonventre and Yang 2011). Cellular damage arises during ischemia as a consequence of energy depletion. Paradoxically, upon restoration of blood flow, further cellular injury occurs due to the overproduction of reactive oxygen species (ROS) (Wu et al. 2018).

Comparative biology may uncover protective mechanisms that could be applied to human diseases. Hibernators are naturally protected from is I-R injury, as they survive through repeated cycles of torpor, characterized by dramatic reductions in heart and respiratory rates, followed by interbout arousals where these functions are restored (Carey et al. 2003; Storey and Storey 2010; Joles et al. 2015). Although most hibernators are rodents, which are phylogenetically distant from humans, studies have documented hibernation in primates that are more closely related to humans, such as the Madagascan fat-tailed dwarf lemur *(Cheirogaleus medius)*. Interestingly, unlike other hibernators, primate hibernators do not experience pronounced decreases in body temperature during torpor (Blanco et al. 2018; Dausmann et al. 2004). Therefore, investigating the molecular mechanisms that regulate hibernation is of particular interest, as it may lead to the development of therapeutic strategies against I-R injury.

I-R injury is the most common cause of acute AKI, with renal proximal tubular epithelial cells (RPTECs) being particularly vulnerable due to their high metabolic demands (Bonventre and Yang 2011). In severely ill patients, the onset of AKI significantly increases mortality rates (Uchino et al. 2005). Furthermore, there is a risk of incomplete recovery from AKI, leading to the development of chronic kidney disease (CKD). Notably, even in cases of AKI where full biochemical recovery is achieved, the likelihood of future CKD development remains elevated (Hsu 2012). At the molecular level, I-R injury-induced cell death plays a critical role in the pathogenesis of AKI. Studies using human and mouse RPTEC cultures under conditions of anoxia followed by reoxygenation have demonstrated that cell death occurs via apoptosis and ferroptosis (Eleftheriadis et al. 2018b). Preservation of energy during anoxia is crucial for maintaining cellular integrity. Anoxia leads to the cessation of oxidative phosphorylation, resulting in decreased ATP levels, as confirmed in mouse RPTECs. However, Syrian hamster RPTECs are able to preserve ATP levels during anoxia. Two of the most energy-intensive cellular processes are protein translation and the activity of the Na^+^-K^+^ ATPase. During anoxia, both mouse and Syrian hamster RPTECs downregulate protein translation. However, the activity of the Na^+^-K^+^ ATPase in Syrian hamster RPTECs is lower than in mouse RPTECs, and is preserved under anoxia, likely due to reduced sodium and potassium leakage across the cell membrane through the associated channels (Eleftheriadis et al. 2018a). During reoxygenation, excessive ROS production causes DNA damage, triggering a DNA damage response that leads to apoptosis in human RPTECs (Eleftheriadis et al. 2022). Additionally, ROS-induced lipid peroxidation under these conditions results in ferroptosis (Eleftheriadis et al. 2022), a process also observed in isolated mouse renal tubules (Linkermann et al. 2014b). In contrast, RPTECs derived from the naturally hibernating Syrian hamster *(Mesocricetus auratus)* are resistant to both anoxia and reoxygenation (Eleftheriadis et al. 2018b). Studies have shown that cells and organs of native hibernators resist I-R injury-induced cell death, and various molecular mechanisms have been proposed to confer protection against apoptosis and ferroptosis (Dave et al. 2006; Quinones et al. 2016; Bogren et al. 2014; Bogren and Drew 2014; Logan and Storey 2017; Eleftheriadis et al. 2019a, b). Numerous hibernating species that have been studied exhibit protection against ischemia-reperfusion injury, not only in the kidney but also in various other organs (Jiang et al. 2019; Talaei et al. 2011; Kurtz et al. 2006; Otis et al. 2017; Saleem et al. 2021; Frerichs and Hallenbec 1998; Zancanaro et al. 1999). Interestingly, the mechanisms that protect native hibernators from I-R injury may also confer protection against other harmful stimuli that elevate ROS production. For example, thirteen-lined ground squirrels demonstrate resistance to cisplatin-induced AKI (Jain and Jani 2020), and experimental evidence further indicates that native hibernators are protected against radiation-induced injury (Puspitasari et al. 2021).

In addition to cell death, cellular senescence is another critical response to injury that significantly influences the progression of AKI (Canaud and Bonventre 2014; Docherty et al. 2019; Yan et al. 2016; Yu and Bonventre 2020). Senescent cells are characterized by a permanent cell-cycle arrest, even when conditions are favorable for cell-cycle progression. Moreover, they acquire a senescence-associated secretory phenotype (SASP), leading to the continuous production of various proinflammatory and profibrotic cytokines that adversely affect neighboring healthy cells. In the context of AKI, senescent tubular epithelial cells lose the ability to dedifferentiate and proliferate, which impairs their capacity to replace adjacent dead cells, thereby reducing the likelihood of recovery and contributing to the development of CKD. Additionally, the secretion of proinflammatory and profibrotic cytokines by senescent cells may not only accelerate the progression of existing CKD but also increase the risk of developing new CKD in the future (Canaud and Bonventre 2014; Docherty et al. 2019; Yan et al. 2016; Yu and Bonventre 2020).

In human RPTECs, the burst of ROS induced by I-R causes DNA damage, which triggers a DNA damage response (DDR) leading to cellular senescence (Eleftheriadis et al. 2022). Surprisingly, the occurrence of cellular senescence has not been sufficiently investigated in cells derived from native hibernators. In this study, we aimed to assess whether anoxia-reoxygenation, a condition that mimics I-R, triggers a DDR and ultimately induces senescence in primary RPTECs of the native hibernator, the Syrian hamster. For comparison, primary RPTECs derived from the phylogenetically related mouse were used as controls. Mice under certain conditions experience daily torpor. However, hibernation differs from daily torpor not only in duration but also in several critical aspects, including the absolute values and extent of metabolic depression, the mechanisms that induce metabolic depression, and, importantly, the degree of circulatory collapse. The latter plays a significant role in organ ischemia during both hibernation bout and torpor (Geiser 2004; Swoap and Gutilla 2009).

## Materials and methods

### Cell culture conditions

Primary Syrian hamster RPTECs (cat. no. HM-6015, Cell Biologics, Chicago, IL, USA) and C57BL/6 mouse RPTECs (cat. no. C57-6015, Cell Biologics) were cultured in Complete Epithelial Cell Medium, supplemented with an epithelial cell growth supplement, fetal bovine serum, and antibiotics (cat. no. M6621, Cell Biologics). According to the manufacturer’s instructions, the Epithelial Cell Medium supplement kit, containing 0.5 mL insulin-transferrin-selenium, 0.5 mL epithelial growth factor, 5 mL L-glutamine, 5 mL antibiotic-antimycotic solution, and 10 mL fetal bovine serum, was added to 500 mL of basal medium prior to use. Cells were expanded in 75 cm² flasks, and passage two cells were used for the experiments.

Cells were seeded in 96-well plates (10⁴ cells/well) or 6-well plates (3 × 10⁵ cells/well) and incubated for 12 h at 37 °C before the onset of anoxia. Cell confluency, assessed by inverted microscopy, was consistent across all experiments at the start. To induce anoxia, the GasPak™ EZ Anaerobe Container System with Indicator (cat. no. 26001, BD Biosciences, S. Plainfield, NJ, USA) was employed, reducing oxygen concentration to less than 1%. These anoxic conditions, simulating ischemia, were maintained for 24 h.

Subsequently, cells were washed with Dulbecco’s phosphate-buffered saline (PBS) (Sigma-Aldrich; Merck Millipore, Darmstadt, Germany), the culture medium was replenished, and the cells were incubated under reoxygenation conditions in a humidified atmosphere containing 5% CO₂ at 37 °C for 2 h, mimicking reperfusion.

The durations of anoxia and reoxygenation were selected based on a prior study from our laboratory, which demonstrated that primary Syrian hamster RPTECs are highly resistant to both anoxia and reoxygenation, whereas primary mouse RPTECs significantly decline after 48 h of anoxia or 4 h of reoxygenation (Eleftheriadis et al. 2018b). In this study, to ensure cell viability, the cells were exposed to anoxic conditions or reoxygenation for half of the previously specified durations, specifically 24 h of anoxia followed by 2 h of reoxygenation. All experiments were performed in triplicate.

### Assessment of the cellular protein of interest

Hamster and mouse RPTECs were cultured in 6-well plates as previously described. Following the reoxygenation period, the cells were lysed using the T-PER tissue protein extraction reagent (Thermo Fisher Scientific Inc., Waltham, MA, USA) supplemented with protease and phosphatase inhibitors (Sigma-Aldrich; Merck Millipore and Roche Diagnostics, Indianapolis, IN, USA, respectively). Protein concentration was determined using the Bradford assay (Sigma-Aldrich; Merck Millipore), and 10 µg of protein from each sample was utilized for western blot analysis.

For western blotting, 4–12% bis-tris acrylamide gels (NuPAGE 4–12% Bis-Tris Gel, 1.0 mm x 15 well, Invitrogen; Thermo Fisher Scientific, Inc.) were employed. After electrophoresis, proteins were transferred to polyvinylidene difluoride (PVDF) membranes, which were incubated with primary antibodies for 16 h at 4 °C, followed by incubation with secondary antibodies for 30 min at room temperature. When reprobing of PVDF blots was necessary, the Restore Western Blot Stripping Buffer (Thermo Fisher Scientific Inc.) was used. Since the PVDF membranes were reprobed, the β-actin loading control remains consistent across all images generated from the protein analysis on the same gel.

Primary antibodies included 4-Hydroxynonenal (4-HNE, 1:500, cat. no. ab46545, Abcam, Cambridge, UK), phosphorylated histone H2AX at Ser139 (γ-H2AX, 1:500, cat. no. NB100-2280, Novus Biologicals, Abingdon, Oxon, UK), ataxia telangiectasia mutated kinase (ATM, 1:1000, cat. no. 2873, Cell Signaling Technology, Danvers, MA, USA), phosphorylated ATM at Ser1981 (p-ATM, 1:1000, cat. no. 5883, Cell Signaling Technology), tumor suppressor p53 (p53, 1:1000, cat. no. 2524, Cell Signaling Technology), phosphorylated p53 at Ser15 (p-p53, 1:1000, cat. no. 9284, Cell Signaling Technology), p21 Waf1/Cip1 (p21, 1:1000, cat. no. 37543, Cell Signaling Technology), Ki-67 (1:1000, cat. no. NBP2-22112, Novus Biologicals), and GLB-1 (β-galactosidase, 1:500, cat. no. ab55176, Abcam). Secondary antibodies included the anti-rabbit IgG, HRP-linked antibody (1:1000, cat. no. 7074, Cell Signaling Technology) and the anti-mouse IgG, HRP-linked antibody (1:1000, cat. no. 7076, Cell Signaling Technology).

Detection of the western blot bands was achieved using the LumiSensor Plus Chemiluminescent HRP Substrate kit (GenScript Corporation, Piscataway, NJ, USA), and densitometric analysis of the bands was performed using ImageJ software (National Institutes of Health, Bethesda, MD, USA). All experiments were repeated three times.

All original western blots are provided as supplementary material entitled “Whole WB images”.

### Assessment of ROS production, cell death and IL-6 production

ROS production was evaluated in RPTECs cultured in 96-well plates. The fluorescent probe CellROX^®^ Deep Red Reagent (cat. no. C10422, Invitrogen, Life Technologies) was added at a concentration of 5 µM and incubated for 30 min at 37 °C. Following incubation, the RPTECs were washed with PBS, and the fluorescence signal was measured using an EnSpire Multimode Plate Reader (Perkin Elmer, Waltham, MA, USA). These experiments were conducted after 2 h of reoxygenation and were repeated three times.

Cell death was assessed using an LDH release assay with the Cytotox Non-Radioactive Cytotoxicity Assay kit (cat. no. G1780, Promega Corporation, Madison, WI, USA). RPTECs were cultured in 96-well plates for this assay. The percentage of cell death was calculated using the following formula: Cell death (%) = (LDH in the supernatant: Total LDH) x 100. Experiments were conducted in triplicate and performed after 2 h of reoxygenation.

Interleukin-6 (IL-6) production was quantified in the supernatants of RPTECs cultured in 6-well plates. The measurement was conducted using the Mouse IL-6 ELISA Kit (cat. no. E-EL-M0044, Elabscience Biotechnology Inc, Houston, TX, USA). These experiments were performed after 2 h of reoxygenation and were repeated three times.

### Statistical analysis

Data were analyzed with the IBM SPSS Statistics for Windows, Version 29 (IBM Corp., Armonk, NY, USA). Paired t-test was used for the comparison of means. Results were presented as mean ± SEM, and a *p* < 0.05 was considered statistically significant.

## Results

### Anoxia-reoxygenation induces ROS production, lipid peroxidation, and cell death in mouse RPTECs, while although ROS production and lipid peroxidation increased in hamster RPTECs, cell death does not occur

**Fig. 1:**
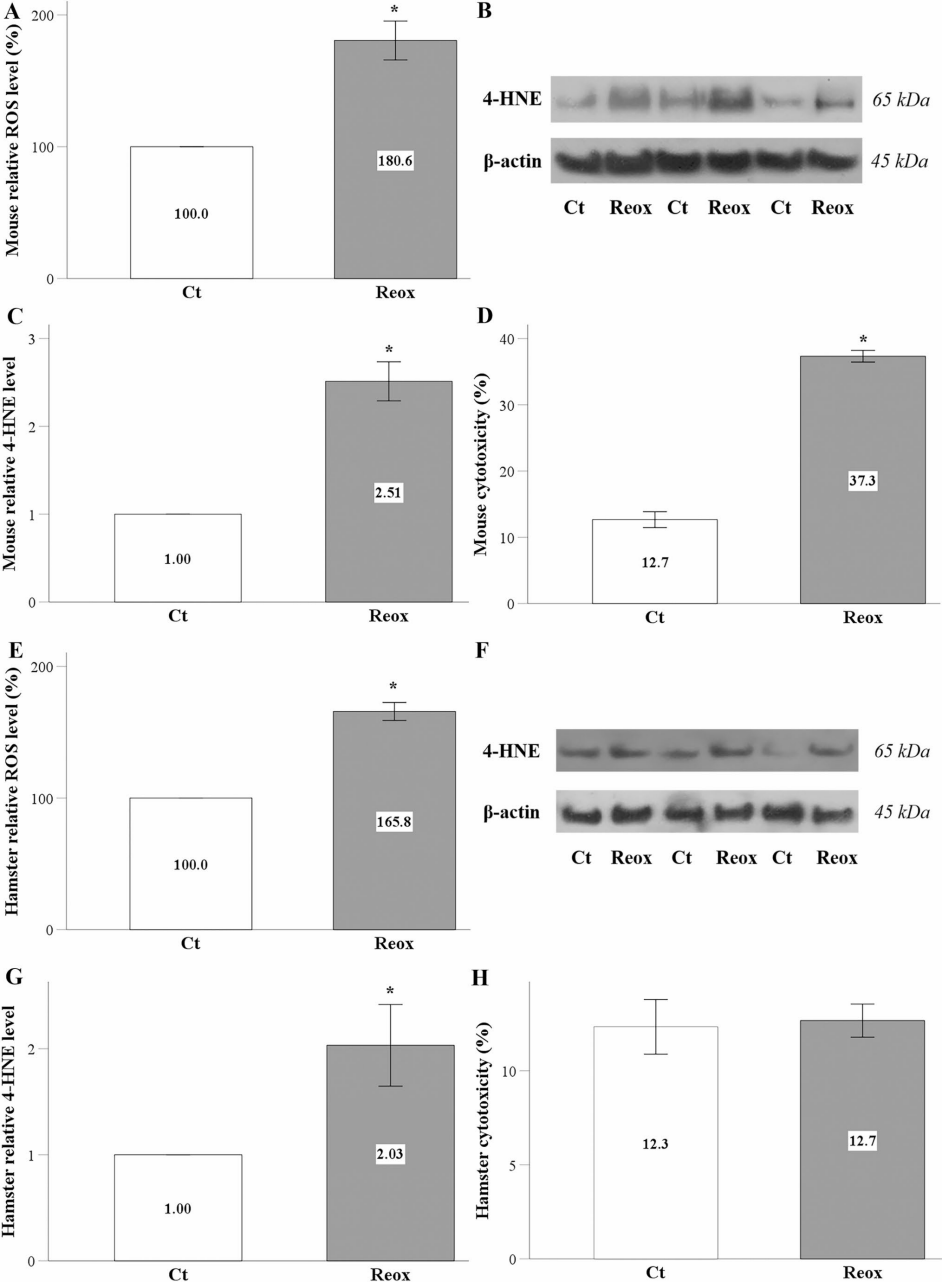
Anoxia-reoxygenation increases ROS and lipid peroxidation in both mouse and hamster RPTECs, but hamster cells survive. Anoxia-reoxygenation increased ROS and 4-HNE-modified proteins in mouse RPTECs (**A**, **B**, and **C**) and induced cell death (**D**). In hamster RPTECs, anoxia-reoxygenation similarly elevated ROS and 4-HNE-modified proteins (**E**, **F**, and **G**), but did not impact cell survival (**H**). White bars correspond to control conditions, while gray bars indicate anoxia-reoxygenation conditions. Error bars represent the standard error of the mean (SEM), and asterisks denote statistical significance with 𝑝<0.05

Anoxia-reoxygenation was found to elevate ROS production in mouse RPTECs (Fig. 1A). This increase in oxidative stress was further validated by the elevated levels of proteins modified by 4-hydroxynonenal (4-HNE), a lipid peroxidation end-product, in mouse RPTECs subjected to anoxia-reoxygenation (Fig. 1B and C). Additionally, the lactate dehydrogenase (LDH) release assay indicated that anoxia-reoxygenation led to cell death in mouse RPTECs (Fig. 1D).

Similarly, anoxia-reoxygenation also enhanced oxidative stress in hamster RPTECs, as demonstrated by the increased ROS production (Fig. 1E) and higher levels of 4-HNE-modified proteins (Fig. 1F and G). However, in contrast to mouse RPTECs, anoxia-reoxygenation did not induce cell death in hamster RPTECs (Fig. 1H).

### Anoxia-reoxygenation elicits a DDR in mouse but not in hamster RPTECs

**Fig. 2:**
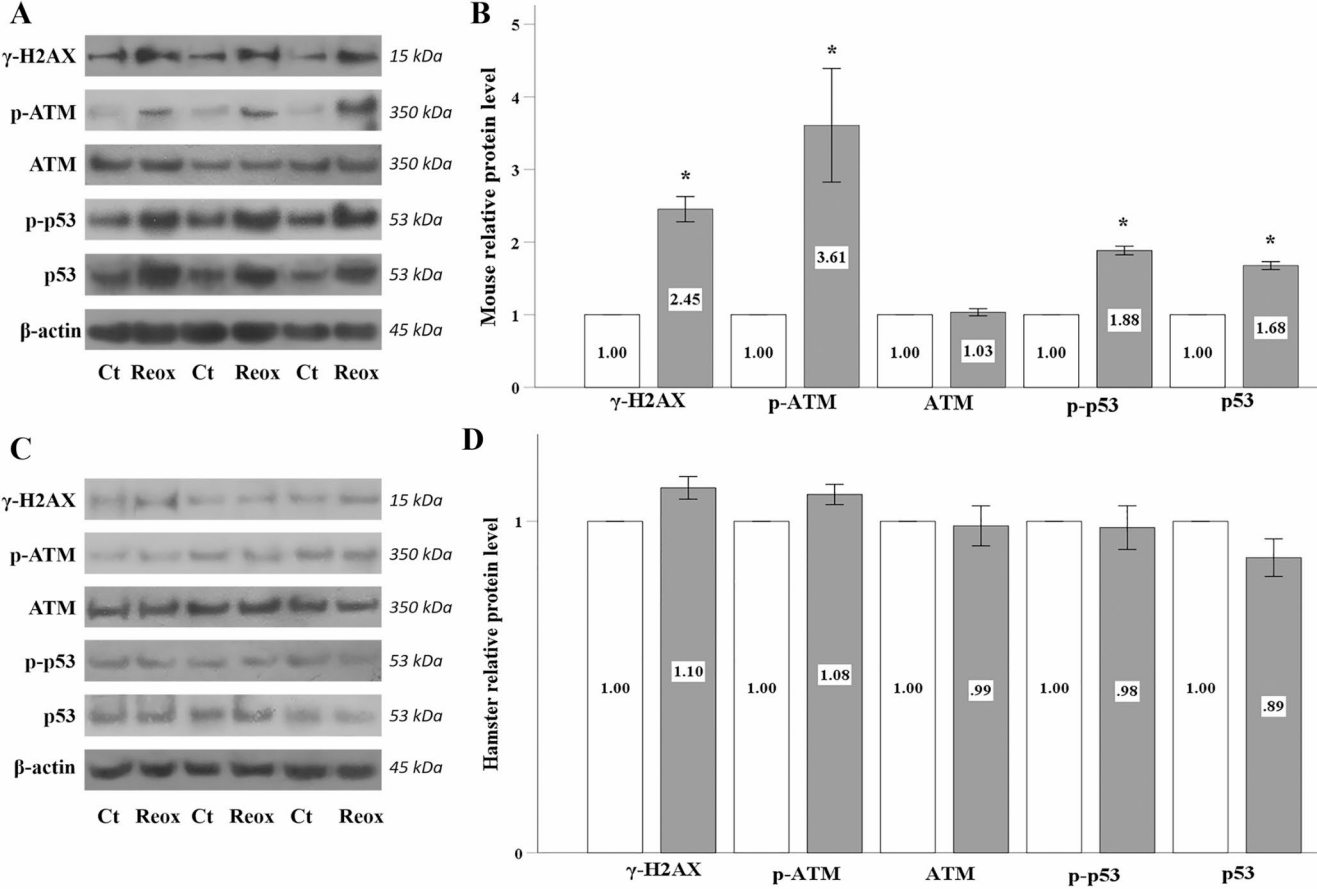
Anoxia-reoxygenation elicits a DDR in mouse but not in hamster RPTECs. In mouse RPTECs, anoxia-reoxygenation triggered a DDR, as indicated by elevated levels of γH2AX, p-ATM, p-p53, and total p53 (**A** and **B**). In contrast, anoxia-reoxygenation did not induce a DDR in hamster RPTECs, as the levels of γH2AX, p-ATM, p-p53, and p53 remained unchanged (**C** and **D**). White bars correspond to control conditions, while gray bars indicate anoxia-reoxygenation conditions. Error bars represent the standard error of the mean (SEM), and asterisks denote statistical significance with 𝑝<0.05

In mouse RPTECs, anoxia-reoxygenation triggered a DNA damage response (DDR), as evidenced by the increased levels of γH2AX, phosphorylated ATM (p-ATM), phosphorylated p53 (p-p53), and eventually p53 (Fig. 2A and B).

In contrast, anoxia-reoxygenation did not elicit a DDR in hamster RPTECs, as the levels of γH2AX, p-ATM, p-p53, and p53 remained stable (Fig. 2C and D).

### Anoxia-reoxygenation induces cellular senescence in mouse but not in hamster RPTECs

**Fig. 3:**
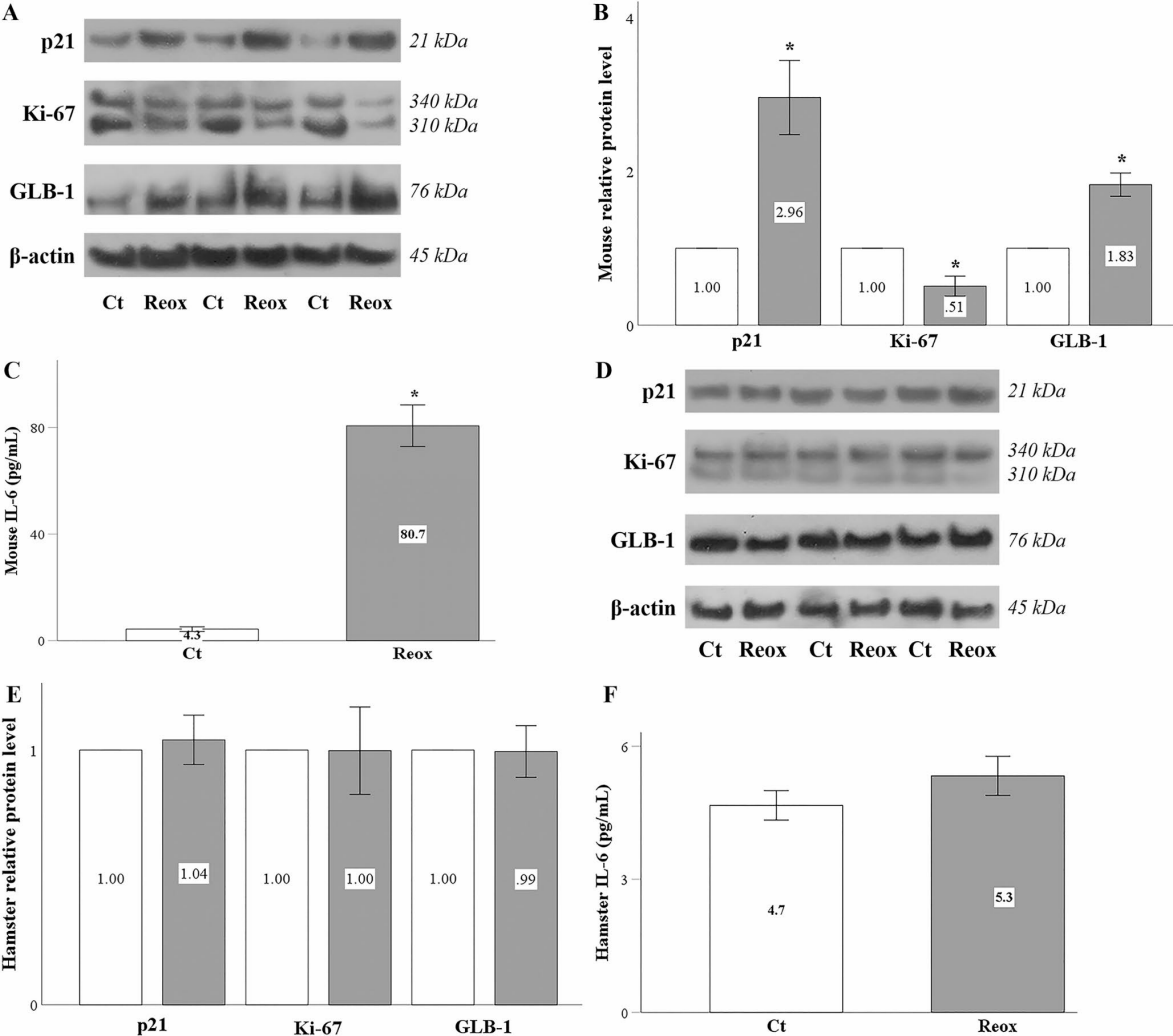
Anoxia-reoxygenation induces cellular senescence in mouse but not in hamster RPTECs. In mouse RPTECs, anoxia-reoxygenation triggered cellular senescence, as indicated by elevated levels of the cell cycle inhibitor p21, reduced expression of the proliferative marker Ki-67, and increased expression of the senescence-associated marker GLB-1 (**A** and **B**), accompanied by enhanced production of IL-6 (**C**).In contrast, anoxia-reoxygenation did not induce cellular senescence in hamster RPTECs, as indicated by the stable levels of p21, Ki-67, GLB-1 and IL-6 production (**D**, **E**, and **F**). White bars correspond to control conditions, while gray bars indicate anoxia-reoxygenation conditions. Error bars represent the standard error of the mean (SEM), and asterisks denote statistical significance with 𝑝<0.05

In mouse RPTECs, anoxia-reoxygenation induced cellular senescence, as indicated by the increased level of the cell cycle inhibitor p21, the decreased level of the proliferative marker Ki-67, and the upregulation of the cellular senescence marker β-galactosidase (GLB-1) (Fig. 3A and B). Furthermore, mouse RPTECs subjected to anoxia-reoxygenation acquired a senescence-associated secretory phenotype (SASP), characterized by the significantly elevated production of IL-6 (Fig. 3C).

On the contrary, anoxia-reoxygenation did not induce cellular senescence in hamster RPTECs, as evidenced by the stable levels of p21, Ki-67, GLB-1, and IL-6 production (Fig. 3D and E, and 3F).

## Discussion

Studying the molecular mechanisms underlying I-R injury is of great importance, as I-R injury is implicated in numerous human diseases and is the most common cause of AKI (Neri et al. 2017; Bakthavachalam and Shanmugam 2017; Tsukamoto et al. 2010; Bonventre and Yang 2011). RPTECs are particularly susceptible to I-R injury, and their death can lead to the onset of AKI (Bonventre and Yang 2011). Moreover, cellular senescence plays a significant role in determining whether recovery from AKI is achieved or if CKD is established, and it may also contribute to the development of CKD in the future, even after apparent full recovery (Canaud and Bonventre 2014; Docherty et al. 2019; Yan et al. 2016; Yu and Bonventre 2020). In renal tubules I-R induces oxidative stress, triggers a DDR, and ultimately leads to cell death through apoptosis or ferroptosis, or to cellular senescence (Schumer et al. 1992; Linkermann et al. 2014a, b; Garg and Vucic 2016; Canaud and Bonventre 2014; Docherty et al. 2019; Yan et al. 2016; Yu and Bonventre 2020). Similar outcomes have been observed under controlled conditions in RPTEC cultures subjected to anoxia-reoxygenation, a model that simulates I-R (Eleftheriadis et al. 2022).

Extensive knowledge has been acquired regarding the molecular mechanisms involved in the pathogenesis of I-R injury-induced acute kidney injury (AKI) (Schumer et al. 1992; Linkermann et al. 2014a, b; Garg and Vucic 2016; Canaud and Bonventre 2014; Docherty et al. 2019; Yan et al. 2016; Yu and Bonventre 2020). However, there is currently no effective treatment available to prevent or cure this condition. In this study, we evaluated whether anoxia-reoxygenation triggers a DDR that ultimately leads to cellular senescence in cultures of primary RPTECs derived from the hibernator Syrian hamster. Mouse primary RPTEC cultures were used as controls. To simulate I-R conditions, RPTECs were subjected to anoxia, washed with PBS, and then reoxygenated in fresh culture medium. While cells and organs of hibernating mammals exhibit remarkable resistance to I-R-induced cell death (Carey et al. 2003; Storey and Storey 2010; Joles et al. 2015; Dave et al. 2006; Quinones et al. 2016; Bogren et al. 2014; Bogren and Drew 2014; Logan and Storey 2017; Eleftheriadis et al. 2019a, b), the impact of I-R on cellular senescence in such species has not been thoroughly investigated. Exploring the effects of I-R on hibernators may uncover new therapeutic strategies with potential clinical applications.

As expected, in mouse RPTECs subjected to anoxia-reoxygenation, there was a notable increase in ROS production, as evidenced by direct ROS measurements and elevated levels of 4-HNE, a marker of lipid peroxidation (Li et al. 2022). Similar increases in ROS production and 4-HNE levels were observed in hamster RPTECs as well. It is generally believed that mitochondria are the primary source of ROS overproduction during the reperfusion/reoxygenation phase (Granger and Kvietys 2015; Eleftheriadis et al. 2022; Pissas et al. 2024; de Veij Mestdagh et al. 2023). During anoxia, oxidative phosphorylation ceases, but the Krebs cycle continues to generate the electron donors NADH and FADH_2_. I-R may also cause disarray in the electron transport chain (ETC) supercomplexes facilitating electron leakage (Jang et al. 2017; Pissas et al. 2024). When oxygen is restored, the excess electrons available can overwhelm the capacity of the ETC, resulting in electron leakage and subsequent superoxide formation (Granger and Kvietys 2015; Pissas et al. 2024). Another potential mechanism for mitochondrial ROS overproduction during anoxia-reoxygenation involves reverse electron transport at the level of succinate dehydrogenase, where electrons move in the opposite direction through the ETC, contributing to ROS generation (Chouchani et al. 2016; Andrienko et al. 2017). Other significant sources of ROS production are xanthine oxidoreductase, NADPH oxidase, and nitric oxide synthase (Granger and Kvietys 2015).

In contrast to mouse RPTECs, RPTECs from the hibernator Syrian hamster demonstrated resistance to cell death induced by anoxia-reoxygenation, consistent with previous studies. This resistance has been studied extensively in both in vivo and in vitro models, revealing several protective mechanisms. One key mechanism involves the establishment of a new balance between apoptotic and anti-apoptotic proteins, which may protect hibernator cells from apoptosis (Logan and Storey 2017; Jiang et al. 2019; Fleck and Carey 2005; Treat et al. 2023). The preservation of cellular ATP content in hamster RPTECs subjected to anoxia also contributes to cell survival (Eleftheriadis et al. 2018a). Additionally, the overexpression of antioxidant factors appears to provide protection against ferroptosis (Eleftheriadis et al. 2019a, b).

ROS overproduction can lead to double-strand DNA breaks and trigger a DDR. The MRE11-RAD50-NBS1 (MRN) complex plays a crucial role in recognizing DNA damage, recruiting ATM to the site, and facilitating ATM autophosphorylation. This process results in the dissociation of ATM dimers into single molecules with enhanced kinase activity (Dupre et al. 2006). One of the primary targets of activated ATM is histone H2AX. Phosphorylation of H2AX results in γ-H2AX, a well-established marker of DNA damage that is essential for assembling DNA repair proteins and activating cell-cycle checkpoint proteins (Podhorecka et al. 2010). Another target of ATM is p53. Phosphorylation of p53 leads to the dissociation of the E3 ubiquitin ligase Mdm2, preventing p53 proteasomal degradation and allowing its accumulation (Brady and Attardi 2010). These key DDR events were observed in mouse RPTECs subjected to anoxia-reoxygenation. In contrast, no such changes were detected in hamster RPTECs, despite a ROS burst occurring.

Once p53 accumulates, it can lead to either cell-cycle arrest or apoptosis (Brady and Attardi 2010). Cell-cycle arrest is associated with cellular senescence (Kumari and Jat 2021; Hernandez-Segura et al. 2018; Muthamil et al. 2024). p53 promotes cell-cycle arrest by upregulating the expression of the cyclin-dependent kinase inhibitor p21 (Brady and Attardi 2010). In mouse RPTECs subjected to anoxia-reoxygenation, increased p53 levels were associated with elevated p21 expression. In parallel, there was a notable decrease in Ki-67, a marker of proliferation (Schlüter et al. 1993). The observed increase in p53 and p21, along with a decrease in Ki-67, may simply indicate cell cycle arrest. To address this, we additionally assessed the senescence marker GLB-1 and the production of the pro-inflammatory cytokine IL-6, indicative of the acquisition of a SASP phenotype. GLB-1 levels showed a significant increase. This enzyme corresponds to senescence-associated β-galactosidase and, while it may not directly drive the associated process (Lee et al. 2006), it is regarded as a marker of cellular senescence due to its upregulation (Hernandez-Segura et al. 2018; Muthamil et al. 2024). In addition to cell-cycle arrest, senescent cells acquire a SASP phenotype and produce proinflammatory cytokines (Kumari and Jat 2021; Hernandez-Segura et al. 2018; Muthamil et al. 2024). Indeed, in mouse RPTECs exposed to anoxia-reoxygenation, there was a significant increase in IL-6 production. In contrast, none of these changes were observed in hamster RPTECs, suggesting that the cells of this hibernator are resistant to anoxia-reoxygenation-induced cellular senescence. Interestingly, I-R injury has been shown to induce both cell death and cellular senescence simultaneously. This phenomenon has been observed in vivo in experimental models of I-R-induced AKI (Baisantry et al. 2019), as well as in asynchronous cell cultures (Small et al. 2013; Kurosaki et al. 2022). The latter observation might be attributed to variations in the cell cycle stages of cultured cells, as the phase of the cell cycle significantly influences the DDR (Branzei and Foiani 2008; Giunta et al. 2010).

Considering the significant role of senescence in AKI, the resistance of hibernator hamster RPTECs to anoxia-reoxygenation-induced senescence likely contributes to AKI prevention during the repeated I-R cycles associated with torpor-interbout arousals in hibernation. The exact mechanisms underlying this resistance in hamster RPTECs remain to be fully elucidated. One potential mechanism may involve the hamster RPTECs response to high ROS production. Unlike mouse RPTECs (Eleftheriadis et al. 2020), hamster RPTECs upregulate H_2_S-producing enzymes under oxidative stress, leading to increased H_2_S production and subsequent upregulation of the transcription factor Nrf2. Nrf2 enhances the expression of various antioxidant enzymes, such as superoxide dismutase 3 and glutathione reductase, as well as anti-ferroptotic proteins like ferritin H and the cystine-glutamate antiporter (Eleftheriadis et al. 2019b; de Veij Mestdagh et al. 2023). This enhanced response to oxidative stress may aid in preventing or repairing ROS-induced DNA damage. However, further investigation is needed to explore additional mechanisms that contribute to this resistance.

A limitation of our study is its in vitro nature, as findings from in vitro models may not always be directly extrapolated to in vivo conditions. Despite this, our study serves as a foundational step for further research aimed at uncovering the molecular mechanisms behind the resistance of hibernating species to I-R injury-induced cellular senescence. Additionally, the strictly controlled conditions of our in vitro experiments may provide valuable insights into the precise sequence of events that occur in vivo.

In conclusion, RPTECs from the native hibernator Syrian hamster show increased ROS production upon reoxygenation; however, neither DDR nor cellular senescence is observed. Further research is needed to elucidate the precise protective molecular mechanisms involved. Such studies could eventually lead to the development of new therapeutic strategies for I-R injury in non-hibernating species, including humans.

## Electronic supplementary material

Below is the link to the electronic supplementary material.

Supplementary Material 1

## Data Availability

Data supporting the findings of this study are available within the paper and its Supplementary Information.
