# Supplementary material for: In renal proximal tubular epithelial cells of the hibernator Syrian hamster, anoxia-reoxygenation-induced reactive oxygen species bursts do not trigger a DNA damage response and cellular senescence
Source: J Comp Physiol B. 2025 Feb 5;195(1):91–101. doi: 10.1007/s00360-025-01604-5 (PMC11839863; doi:10.1007/s00360-025-01604-5)
Supplement: Supplementary file 1 — Supplementary Material 1 [file 360_2025_1604_MOESM1_ESM.pdf]

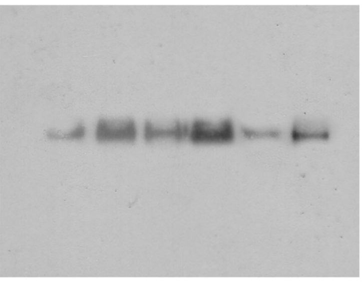

Mouse 4-HNE modified protein
(corresponds to Fig.1B, 1st lane)

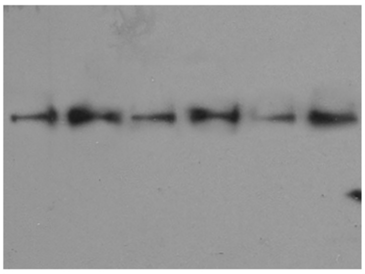

Mouse γ-H2AX
(corresponds to Fig.2A, 1st lane)

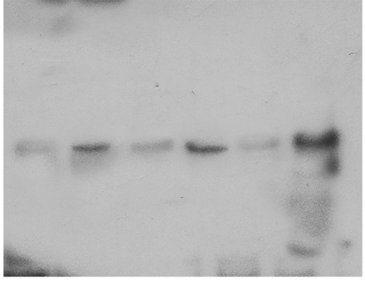

Mouse p-ATM
(corresponds to Fig.2A, 2nd lane)

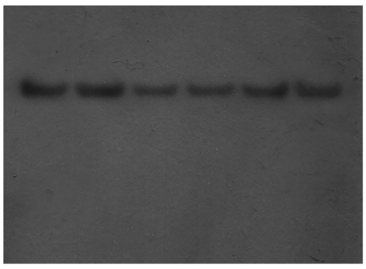

Mouse ATM
(corresponds to Fig.2A, 3rd lane)

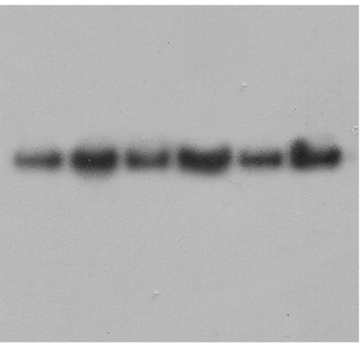

Mouse p-p53
(corresponds to Fig.2A, 4th lane)

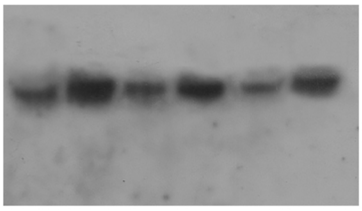

Mouse p53
(corresponds to Fig.2A, 5th lane)

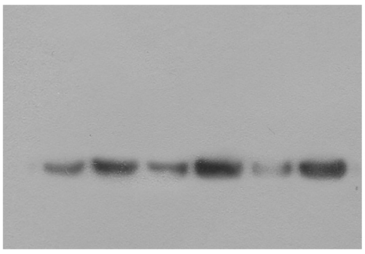

Mouse p21
(corresponds to Fig.3A, 1st lane)

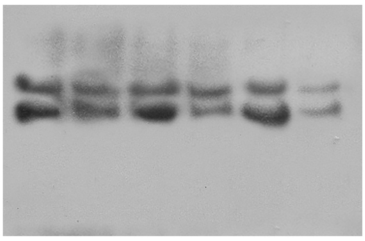

Mouse Ki-67
(corresponds to Fig.3A, 2nd lane)

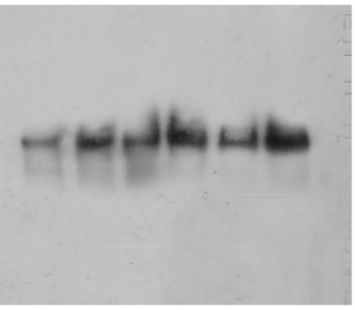

Mouse Glb-1
(corresponds to Fig.3A, 3rd lane

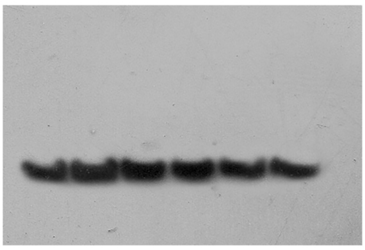

Mouse β-actin 1
(corresponds to Fig.1B, 2nd lane & Fig. 3A, 4th lane)

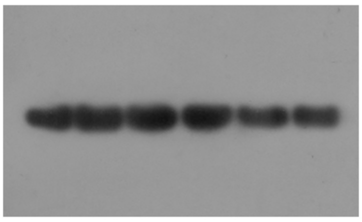

Mouse β-actin 2
(corresponds to Fig.2A, 6th lane)

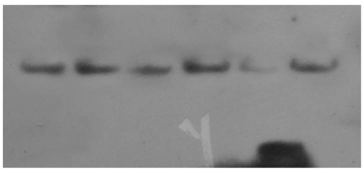

Hamster 4-HNE modified protein
(corresponds to Fig.1F, 1st lane)

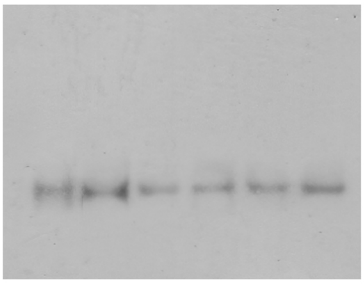

Hamster γ-H2AX
(corresponds to Fig.2C, 1st lane)

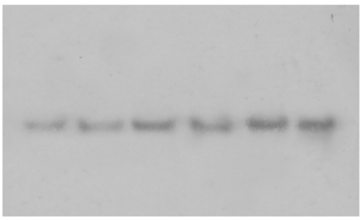

Hamster p-ATM
(corresponds to Fig.2C, 2nd lane)

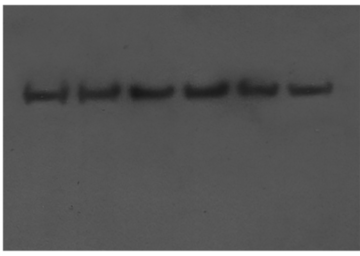

Hamster ATM
(corresponds to Fig.2C, 3rd lane)

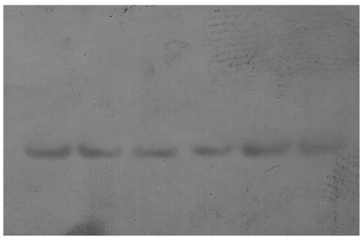

Hamster p-p53
(corresponds to Fig.2C, 4th lane)

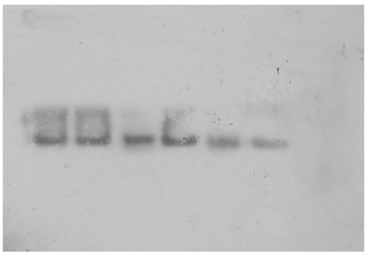

Hamster p53
(corresponds to Fig.2C, 5th lane)

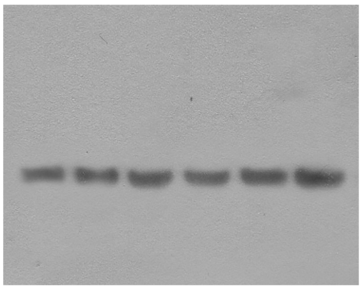

Hamster p21
(corresponds to Fig.3D, 1st lane)

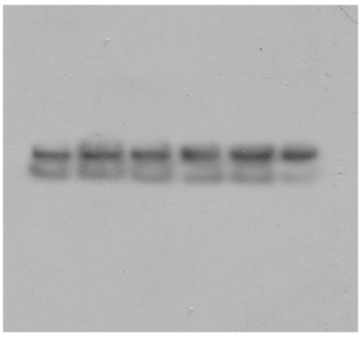

Hamster Ki-67
(corresponds to Fig.3D, 2nd lane)

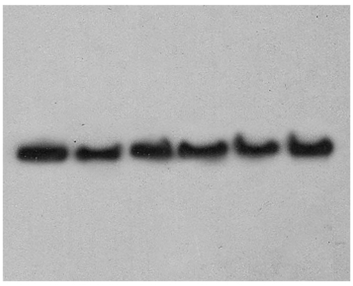

Hamster Glb-1
(corresponds to Fig.3D, 3rd lane)

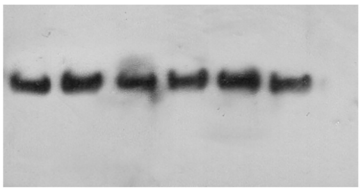

Hamster β-actin 1
(corresponds to Fig.1F, 2nd lane & Fig. 3D, 4th lane)

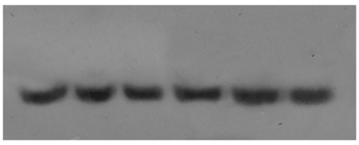

Hamster β-actin 2
(corresponds to Fig.2C, 6th lane)
